# NEAT1 promotes retinoblastoma progression via modulating miR‐124

**DOI:** 10.1002/jcb.28825

**Published:** 2019-04-30

**Authors:** Lufei Wang, Defeng Yang, Rui Tian, Hui Zhang

**Affiliations:** ^1^ Department of Ophthalmology The Second Hospital of Jilin University Changchun Jilin People's Republic of China

**Keywords:** apoptosis, long noncoding RNA, microRNA‐124, nuclear‐enriched abundant transcript 1, proliferation, retinoblastoma

## Abstract

The long noncoding RNA nuclear‐enriched abundant transcript 1 (NEAT1) is reportedly involved in the initiation and progression of cancers of several types. However, the role, expression status, and the detailed mechanism of NEAT1 in retinoblastoma (RB) yet need to be unraveled. We explored the role and the mechanism of NEAT1 activity in RB. Our data show enhanced NEAT1 expression in RB‐affected tissues compared with the corresponding control. Functional experiments reveal that a NEAT1 knockdown in RB cells significantly inhibits proliferation, cycle progression, and facilitates apoptosis and caspase‐3 and ‐9 activities. Besides that, miR‐124 was predicted to be a target of NEAT1 and its reduced expression, as well as the inverse correlation of NEAT1 with miR‐124, was observed in RB‐affected tissues. Further, luciferase and RNA immunoprecipitation (RIP) assays confirmed the interaction between NEAT1 and miR‐124. Rescue experiments confirmed that the inhibition of miR‐124 could reverse the effect of NEAT1 on RB cell proliferation, cycle arrest, apoptosis, and caspase‐3 and ‐9 activities. Thus, NEAT1 promotes RB progression by sponging miR‐124, providing a therapeutic target for RB.

## 1 INTRODUCTION

One of the primary ocular tumors that is malignant is retinoblastoma, which affects the developing retina and is set off by mutations in the retinoblastoma (*RB1*) gene, usually occurring during childhood^1^, ^2^ It is well known that tumorigenesis of several cancers including RB happens chiefly due to oncogene hyperactivation and tumor‐suppressor gene inactivation.^3^ Therefore, exploring the molecular mechanism in the occurrence and progress of RB is important for finding a new and effective treatment for this disease.

A class of RNAs, the long noncoding RNAs (lncRNAs), have lengths bigger than 200 nucleotides, having no protein‐encoding ability,^4^ and have been reported to be involved in numerous biological activities, like cell multiplication, cell apoptosis, cycle arrest, and invasion.^5^ Concurrently, lncRNAs play act as regulators in the occurrence and progress of cancers, and function either as an oncogene or as a tumor‐suppressor gene by regulating tumor‐related genes or pathways.^6^, ^7^ Till date, several cancer‐related lncRNAs have been implicated in RB progression, and act as diagnostic markers and therapeutic target for RB.^8^, ^9^, ^10^

A nuclear‐restricted lncRNA, nuclear paraspeckle assembly transcript 1 (NEAT1), encodes two transcriptional variants, NEAT1‐1 and NEAT1‐2, of lengths 3756 and 22 743 bp, respectively.^11^ NEAT1 participates in several processes, including differentiation of cells,^12^ inflammation^13^ and stress responses.^14^ Mounting evidence indicate that NEAT1 plays key part in tumor initiation and progression in cancers, including breast cancer,^15^ colorectal cancer,^16^ non‐small cell lung cancer,^17^, ^18^ cervical carcinoma,^19^hepatocellular carcinoma,^20^ osteosarcoma,^21^ nasopharyngeal carcinoma,^22^ oral squamous cell carcinoma cells,^23^ ovarian cancer^24^ and esophageal squamous cell carcinoma.^15^ However, more studies are needed to assess the effects of NEAT1 on RB in terms of biological function and the intrinsic mechanisms.

Therefore, this study evaluated the expression levels, function, and the mechanism of NEAT1 activity in RB. We sought to examine the difference in expression of NEAT1 between RB tissues and normal retina tissues. We further investigated in vitro, the effect of the NEAT1 on RB‐affected cells, including the progression of the cell cycle, multiplication, and apoptosis. This study also aims to enhance the knowledge base to discern the RB progression and its mechanism.

## 2 MATERIALS AND METHODS

### 2.1 Tissue sample collection

The Ophthalmology Department in the Second Hospital of Jilin University (Changchun, China) provided us with 32 freshly frozen tissue samples of retinoblastoma and eight tissues samples of normal retina specimens.^8^ The normal retina tissue samples were obtained from ruptured globes. All clinical specimens were obtained from enucleated eyes and flash frozen in liquid N_2_ until use. None of the patients received chemotherapy or local radiotherapy before surgery. This study was carried out as per the Declaration of Helsinki, after acceptance from the Committee of Research Ethics in Second Hospital of Jilin University. Each patient or their parents were informed and their written consent was acquired.

### 2.2 Assays with cultured cells

Three human RB cell lines Y79, WERI‐Rb1, and SO‐RB‐50 ARPE‐19, the epithelial retina cells of human, were purchased from ATCC (Manassas, VA). Each cell line was grown at 37°C in the medium (RPMI‐1640) augmented with 10% fetal bovine serum (both from Gibco, Carlsbad, CA) and antibiotic streptomycin/penicillin (1%; Invitrogen, Waltham, MA) in an atmosphere containing appropriate humidity and 5% CO_2_.

### 2.3 Cell transfection

To assess downregulation of NEAT1, three small interference RNAs (siRNAs), si‐NEAT1#1, #2, and #3 targeting human NEAT1 and its negative control nonsense sequence (si‐NC) were designed and synthesized by GenePharma (Shanghai, China). The miR‐124 inhibitors, its negative control (anti‐miR‐124), mimics of miR‐124 and miR‐NCs (respective negative controls) were brought from Genecopoeia (Guangzhou, China). Y79 cells were transfected for 48 hours with 50 μM of siRNA or 100 nM miR‐124 mimic or inhibitor along with Lipofectamine 2000 (Invitrogen), and the efficiency of transfection was evaluated by quantitative real‐time polymerase chain reaction (qRT‐PCR).

### 2.4 The qRT‐PCR assay

The tissue samples were used for isolating total RNA using the TRIzol reagent (Invitrogen). The total amount and quality of RNA were assessed and for each sample, 1 μg RNA was used for cDNA first strand synthesis with the PrimeScript reverse transcription reagent Kit procured from Takara (Dalian, China), then quantified using SYBR Premix Ex Taq™II (Takara) on a 7500 Fast Real‐Time PCR System (Applied Biosystems, Foster City, CA). The endogenous controls were U6 and glyceraldehyde 3‐phosphate dehydrogenase for normalization to miR‐124 and NEAT1, respectively. The primers used in this study are mentioned (Table 1). The relative gene expression level was determined using the $2^{{-\Delta \Delta C}_{t}}$ formula on ABI software, Foster City, CA.

**Table 1:** Real‐time PCR primers used for NEAT1 or miR‐124 expression analysis

Target genes	Prime (5′‐3′)
U6	F: TCCGATCGTGAAGCGTTC
	R: GTGCAGGGTCCGAGGT
miR‐124	F: GCGCTAAGGCACGCGGT
	R: CAGTGCAGGGTCCGAGGT
NEAT1	F: CTTCCTCCCTTTAACTTATCCATTCAC
	R: CTCTTCCTCCACCATTACCAACAATAC
GAPDH	F: AAGGTGAAGGTCGGAGTCAA
	R: AATGAAGGGGTCATTGATGG

Abbreviations: GAPDH, glyceraldehyde 3‐phosphate dehydrogenase; F, forward; mRNA, messenger RNA; NEAT1, nuclear‐enriched abundant transcript 1; PCR, polymerase chain reaction; R, reverse.

### 2.5 Cell proliferation assay

The viability of the cells was examined via Cell Counting Kit‐8 (CCK‐8 assay, Japan). In brief, the transfected cells at a density of 5 × 10^3^ cells per well were seeded and maintained in 24‐well plate for 24 to 72 hours. In the specified time (24, 48 and 72 hours), 100 μL of fresh medium with 10% CCK‐8 solution was added after removing an equal volume of spent medium, and further maintained at 37°C for 4 hours. Then, the absorbance was detected at 450 nm on an ELISA plate reader (Thermo Labsystems, Helsinki, Finland).

### 2.6 Flow cytometry assay to detect cell cycle arrest

After 48 hours of transfection, the collected cells were centrifuged and suspended again in the binding buffer for assessing the stage of the cell cycle. Using propidium iodide (PI; Beijing Solarbio Science & Technology Co, Ltd, Beijing, China), the cells were stained in the presence of RNase A for 15 minutes. Finally, cell cycle arrest was flow‐cytometrically assessed using BDLSR II from BD Biosciences (Franklin Lakes, NJ), and was evaluated using CellQuest 3.0 software (BD Biosciences).

### 2.7 Apoptosis detection

Cells were collected 48 hours posttransfection and were observed using the Apoptosis Detection Kit Annexin V‐FITC (BD Biosciences), under a flow cytometer (Becton Dickinson, San Jose, CA) as per manufacturer's instruction. The percentage of cell apoptosis was determined using CellQuest 3.0 software (Becton Dickinson).

### 2.8 Assay to determine caspase‐3/9 activities

The caspase‐3/9 activities were measured using Assay Kit AAT‐22820 (Beyotime Institute of Biotechnology, Beijing, China) as per protocol specified by the manufacturer. The absorbance (405 nm) was measured using a microplate reader (BioTek Instruments, Inc, Winooski, VT) to determine caspase‐3/9 activities.

### 2.9 The RNA immunoprecipitation assay

The RNA immunoprecipitation (RIP) assay was performed using the RNA‐Binding Protein Immunoprecipitation Kit (Merck Millipore, Billerica, MA) as per the instructions of the manufacturer. After RIP, qRT‐PCR was done to assess NEAT1 expression as described.

### 2.10 Dual fluorescence assay

Starbase v2.0 software (http://starbase.sysu.edu.cn/) was used to predict the binding sites between miR‐124 and NEAT1. NEAT1 fragments containing the putative or mutated binding sites for miR‐124 were synthesized by GenePharma (Shanghai, China), ligated into psiCHECK2 (Promega Corporation, Madison, WI) and named as WT‐NEAT1 or MT‐NEAT1, respectively. For reporter assays, reporter plasmids (MT‐NEAT1 or WT‐NEAT1) were cotransfected with miR‐124 mimic or miR‐NC in Y79 cells along with Lipofectamine 2000. The activities of luciferases (both Renilla and Firefly) in cell lysates were evaluated 48 hours after transfection using the Dual‐Luciferase Reporter Assay System (Promega Corporation) as per instruction. The data were acquired after Renilla luciferase activity was normalized with the firefly luciferase activity.

### 2.11 Statistical analyses

All results were determined from the experiments that were repeated three times independently and showed as the mean ± SD (standard deviation). SPSS v. 19.0 (IBM Corp, Armonk, NY) was used for all analyses. The Student *t* test and one‐way analysis of variance with Tukey's post‐hoc test were applied to compare among two groups and more than two groups, respectively. The relationship of NEAT1 with miR‐124 was assessed through Spearman's correlation analysis after accounting for the statistically significant difference (*P < *0.05).

## 3 RESULTS

### 3.1 Upregulation of NEAT1 in RB‐affected tissues and cell lines

**Figure 1:**
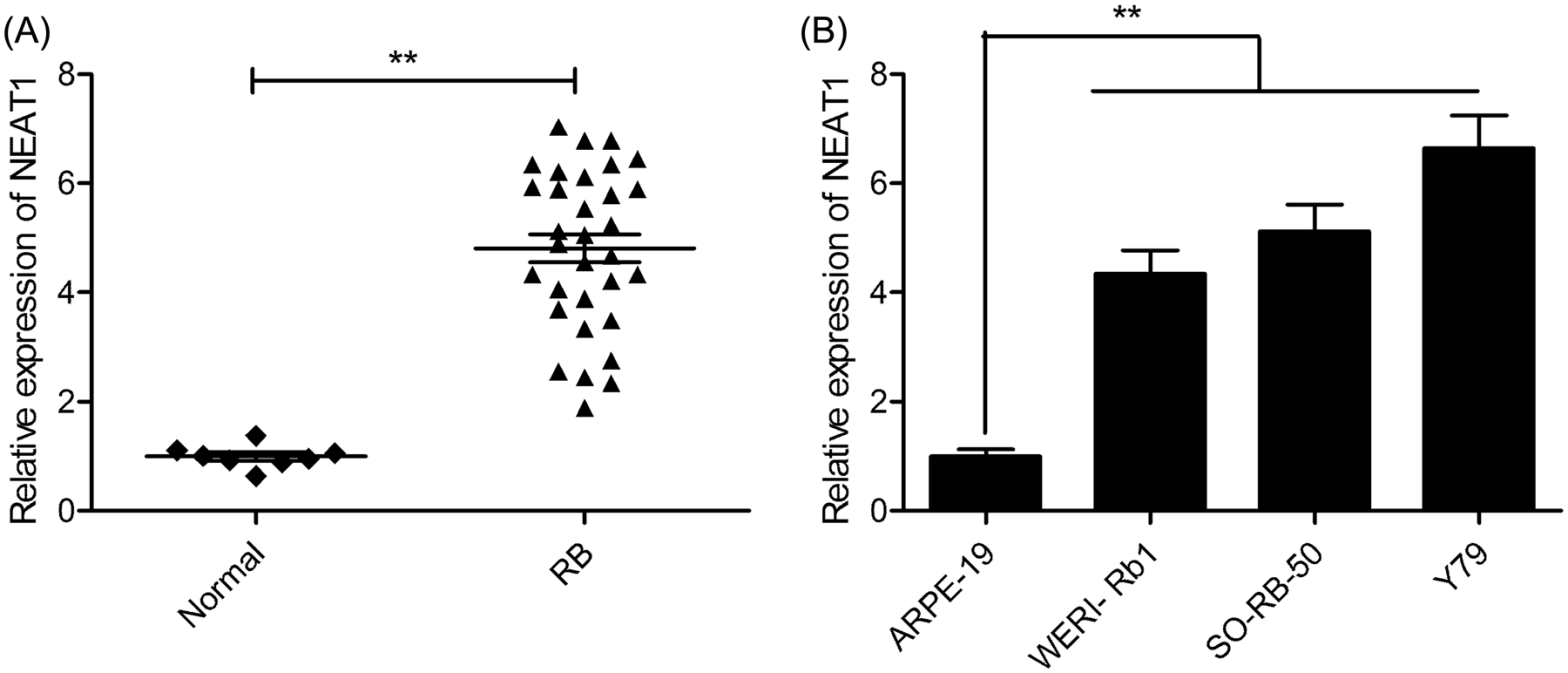
NEAT1 is upregulated in RB tissues and cell lines. A, The expression level of NEAT1 was examined in 32 RB tissues and eight normal retina tissues by qRT‐PCR. B, The expression of NEAT1 was evaluated in the three RB cell lines (WERI‐Rb1, SO‐RB‐50, and Y79) and in human retinal epithelial cells ARPE‐19 by qRT‐PCR. **P < 0.01. NEAT1, nuclear‐enriched abundant transcript 1; qRT‐PCR, quantitative real‐time polymerase chain reaction

When the NEAT1 expression levels in 32 RB‐affected tissues and eight normal retina tissues were assessed by qRT‐PCR, the results show that the RB tissues have significantly higher NEAT1 expression than that in normal retinal tissues (Figure 1A). Subsequently, the NEAT1 expression level in three RB cell lines Y79, WERI‐Rb1, and SO‐RB‐50) and in epithelial cells of the human retina, ARPE‐19 was evaluated (Figure 1B). The three RB cell lines, in comparison to the ARPE‐19 cells, showed significantly upregulated NEAT1 (*P < *0.01). Thus, the genesis of RB may involve the participation of NEAT1.

### 3.2 Knockdown of NEAT1 leads to inhibition of proliferation of RB cell and cell cycle procession

**Figure 2:**
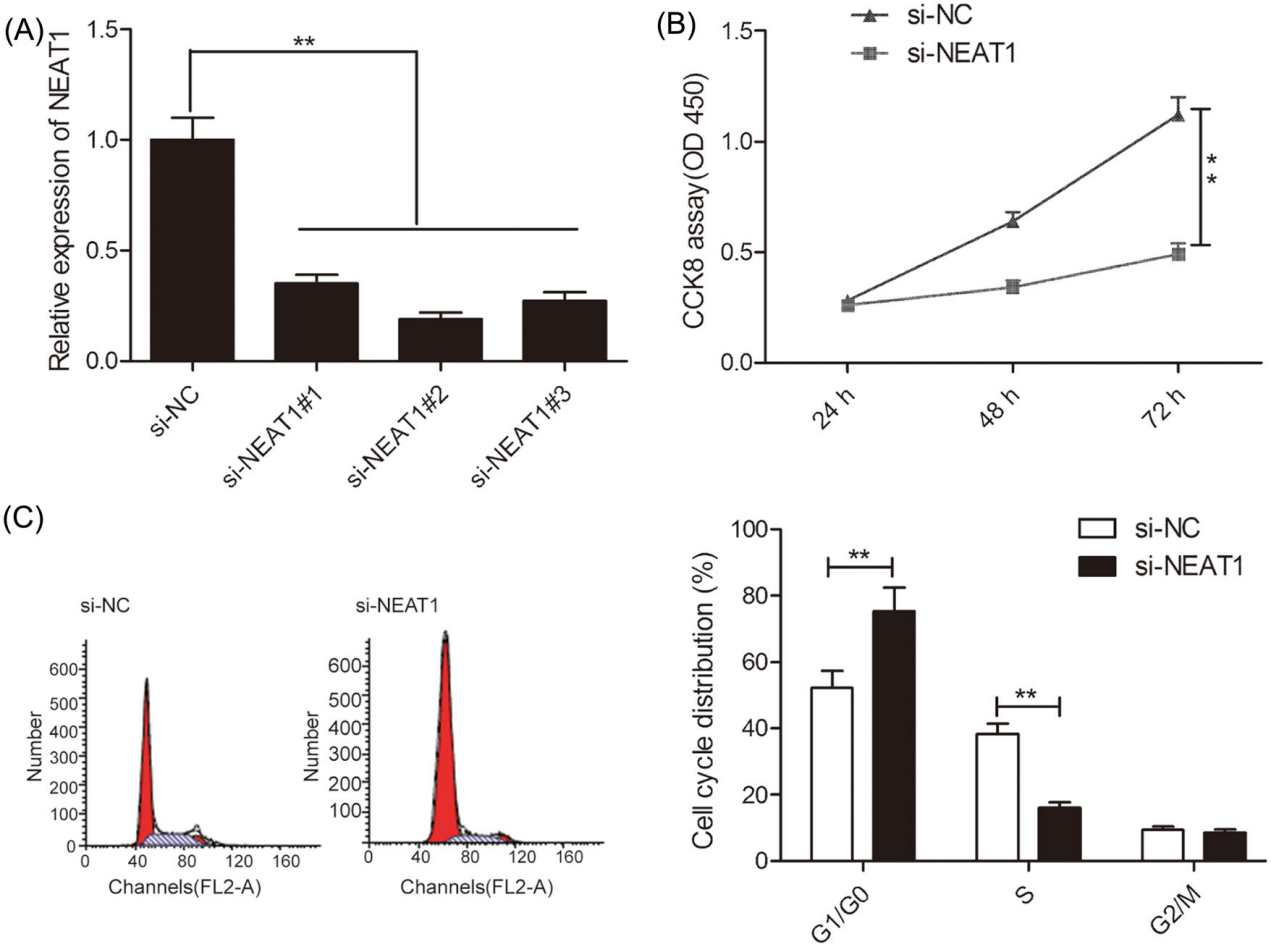
Knockdown of NEAT1 inhibits RB cell proliferation and cell cycle procession. A, The expression of NEAT1 was evaluated in Y79 cells transfected with si‐NEAT1#1, si‐NEAT1#2, and si‐NEAT1#3 by qRT‐PCR. B and C, Cell proliferation and cycle arrest were determined in Y79 cells transfected with si‐NEAT1 or si‐NC. **P < 0.01. NEAT1, nuclear‐enriched abundant transcript 1; qRT‐PCR, quantitative real‐time polymerase chain reaction

To assess the role of NEAT1 in RB cells, the NEAT1 was knocked down using the siRNA against NEAT1, si‐NEAT1#1, si‐NEAT1#2, and si‐NEAT1#3 in Y79 cells. The qRT‐PCR results revealed that the transfection of Y79 cells with the three siRNAs had a notably reduced NEAT1 expression as compared with its levels in the si‐NC‐transfected cells (Figure 2A). The most remarkable effect on NEAT1 expression (81% reduction) was observed through the use of si‐NEAT1#2, as confirmed in Figure 1A. Therefore, si‐NEAT1#2 was selected as the best candidate siRNA for all below studies and was named as si‐NEAT1. In addition, the knockdown of NEAT1 in Y79 cells significantly reduced their viability as observed through the CCK‐8 assay (Figure 2B). To examine the mechanisms and the role of NEAT1 on the of RB cell proliferation, the cell cycle arrest was flow‐cytometrically assessed. The results revealed that NEAT1 knockdown in Y79 cells increased the arrest of the cell cycle at G0/G1 phase and reduced at the S phase (Figure 2D). Thus, in the Y79 cells, NEAT1 knockdown impeded cell proliferation by regulating cell cycle procession.

### 3.3 NEAT1 knockdown induces RB cell apoptosis

**Figure 3:**
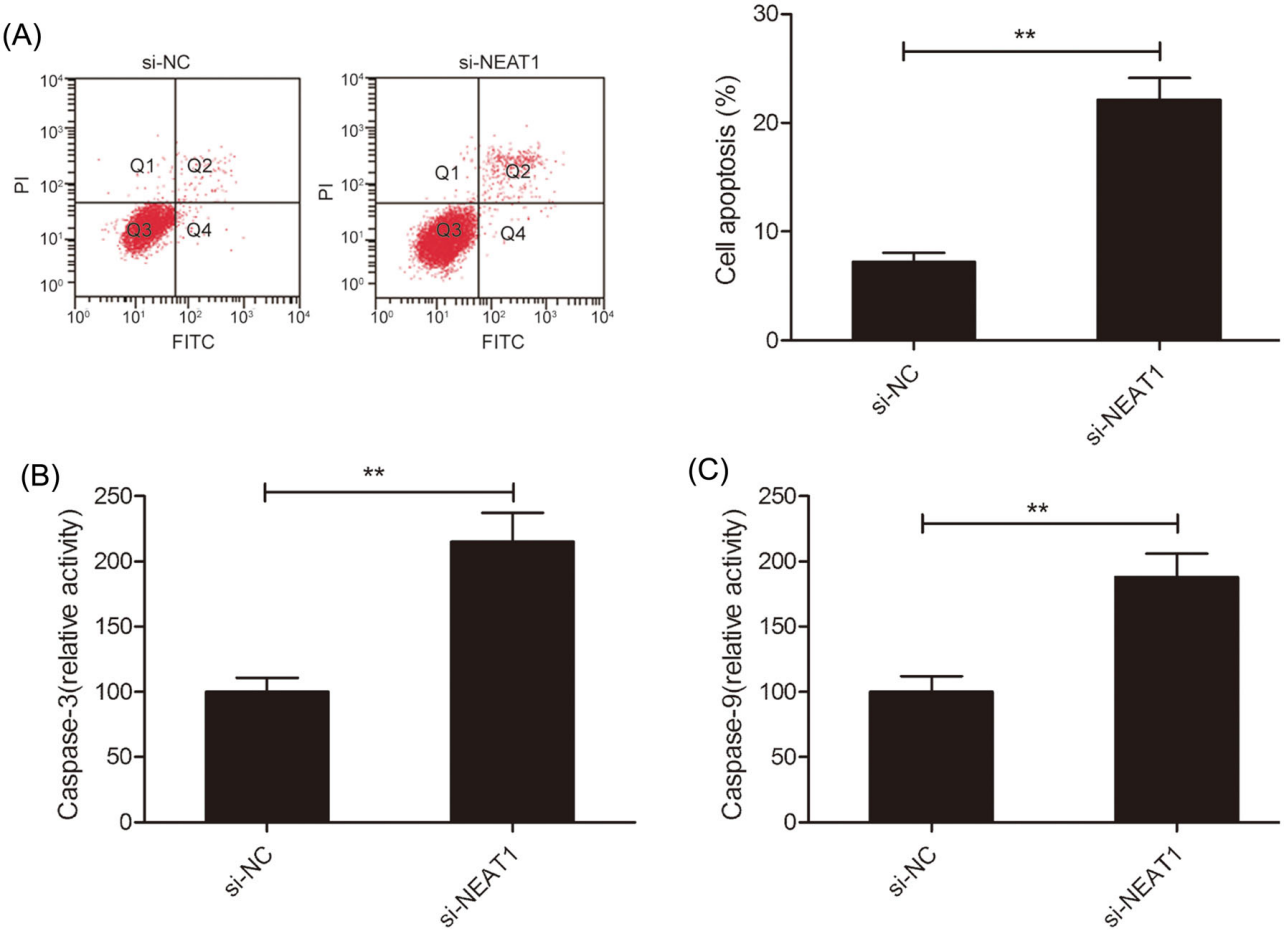
NEAT1 knockdown induces apoptosis of RB cells. A, Cell apoptosis was determined in Y79 cells transfected with si‐NEAT1 or si‐NC by flow cemetery. B and C, The activities of caspase‐3 and ‐9 were measured in Y79 cells transfected with si‐NEAT1 or si‐NC. **P < 0.01. NEAT1, nuclear‐enriched abundant transcript 1; RB, retinoblastoma

The effect of LINC00152 knockdown on the Y79 cell apoptosis was then explored by flow cemetery analysis. As shown in Figure 3A, a remarkable induction in apoptosis in Y79 cells (*P* < 0.01) is observed due to the knockdown of NEAT1. To examine the inherent mechanism of NEAT1 effect cell apoptosis, the activities of caspase‐3 and ‐9 were examined, which were found to be increased notably in the si‐NEAT1‐transfected Y79 cells compared with si‐NC‐transfected cells (Figure 3B and 3C).

### 3.4 miR‐124 expression is directly regulated by NEAT1

**Figure 4:**
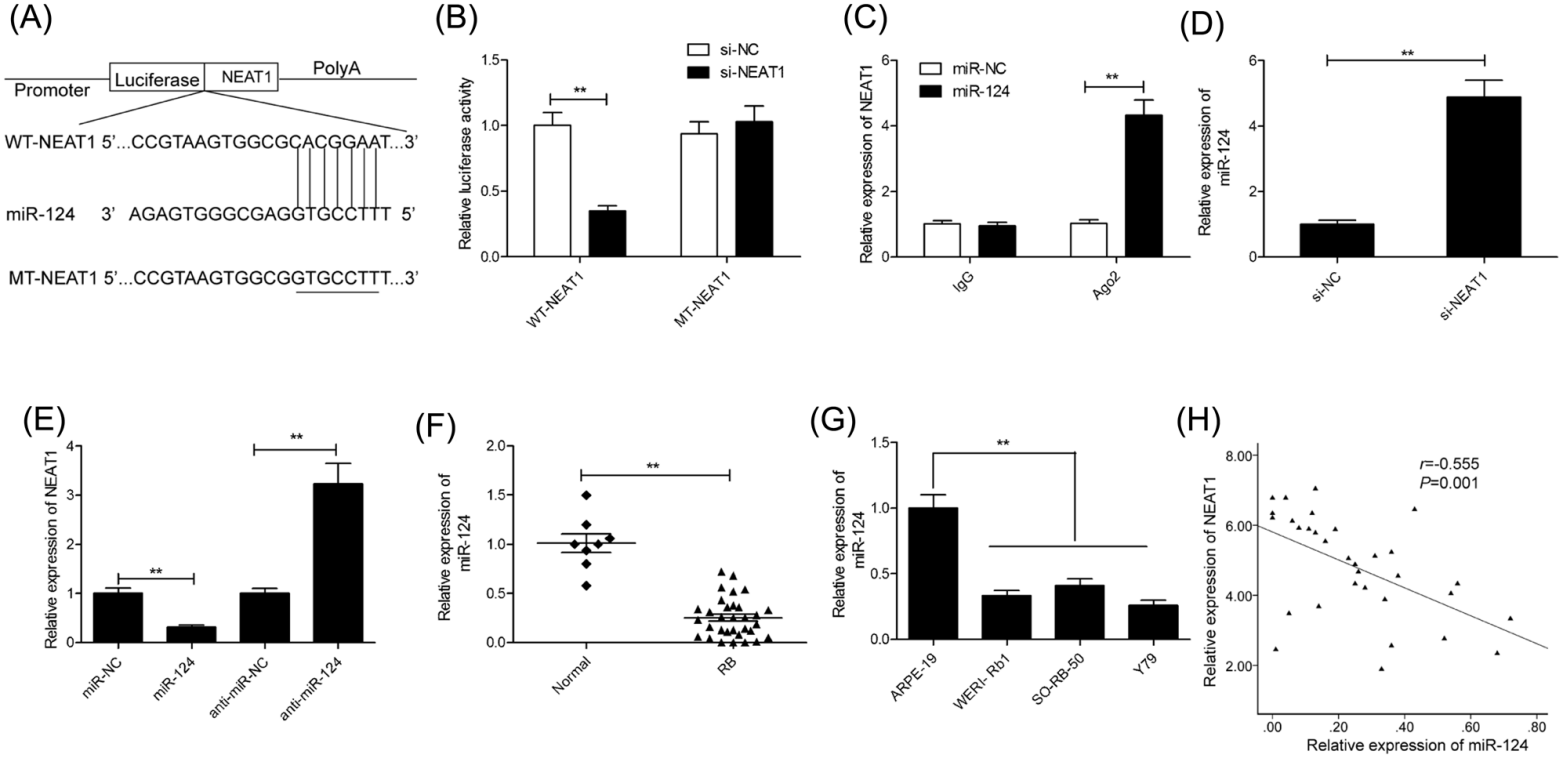
miR‐124 expression is directly regulated by NEAT1. A, miR‐124 and its putative binding sequence in the NEAT1 3′‐UTR. A mutated binding site was generated in the miR‐124 seed region. B, Luciferase activity was measured in Y79 cells cotransfected with miR‐124 mimics or miR‐NC, and luciferase reporter plasmid containing WT‐NEAT1 or MT‐NEAT1. C, The RIP assay with miR‐124 mimics revealed that both miR‐124 and NEAT1 were enriched in Ago2 precipitate compared with IgG in Y79 cells. D, miR‐124 expression levels were examined in Y79 cells transfected with si‐NEAT1 or si‐NC. E, NEAT1 expression levels were measured in Y79 cells transfected with miR‐124 mimics or miR‐124 inhibitor (anti‐miR‐124). F, Expression of miR‐124 in 32 RB tissues and eight normal retina tissues. G, Expression of miR‐124 in the three RB cell lines (WERI‐Rb1, SO‐RB‐50, and Y79) and in human retinal epithelial cells ARPE‐19. H, An inverse association between NEAT1 and miR‐124 expression in RB tissues (n = 32) was identified by Spearman's correlation analysis. **P < 0.01. 3′‐UTR, 3′‐untranslated region; IgG, immunoglobulin G; miR‐124, microRNA‐124; MT, mutant‐type; NEAT1, nuclear‐enriched abundant transcript 1; RIP, RNA immunoprecipitation; WT, wild‐type

LncRNAs reportedly affect the expression of miRNA by acting as a competitive endogenous RNA (ceRNA), thereby influencing tumor progression.^25^ To identify the NEAT1 target miRNAs in RB cells**,** we used a target prediction tool Starbase 2.0, and selected miR‐124 as a probable target (Figure 4A) based on its role in regulating tumor progression.^26^ To confirm this prediction, we carried out luciferase assay and revealed that a significant reduction in the WT‐NEAT1 luciferase activity but not that of MT‐NEAT1 (Figure 4B; *P* < 0.05) as a result of miR‐124 overexpression. Moreover, RIP assay confirmed that both NEAT1 and miR‐124 were enriched in pulled down Ago2 protein in Y79 cells (Figure 4C), indicating that NEAT1 could act as a miR‐124 sponge in RB cells. Concurrently, we found that NEAT1 downregulation in Y79 cells significantly enhanced the miR‐124 levels (Figure 4D; *P* < 0.05), and that mimics of miR‐124 remarkably reduced NEAT1 expression in Y79 cells, while miR‐124 inhibitor obviously increased NEAT1 expression in Y79 cells (Figure 4E; *P* > 0.05). In addition, the miR‐124 was remarkably down‐expressed in RB‐affected tissues and cell lines (Figure 4F and 4G). On Spearman's correlation analysis, an inverse correlation of miR‐124 expression with NEAT1 expression (Figure 4H) was observed, indicating that NEAT1 may reduce miR‐124 availability by binding directly to the latter.

### 3.5 miR‐124 inhibition restores the inhibitory effects of NEAT1 knockdown on RB cells

**Figure 5:**
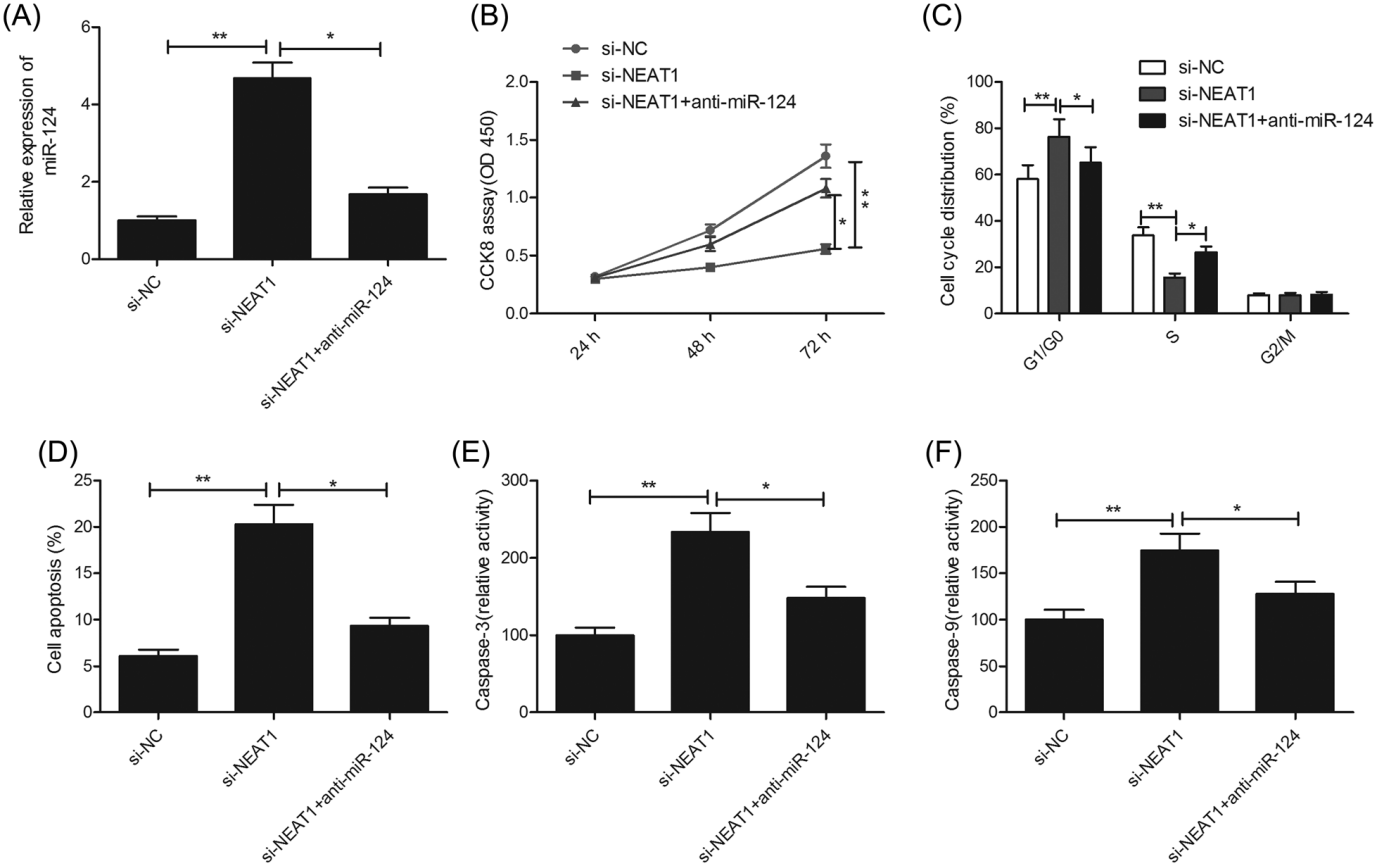
miR‐124 inhibition restores the inhibitory effects of NEAT1 knockdown on RB cells. A, Expression of miR‐124 was examined in Y79 cells after transfection with si‐NC and si‐NEAT1 with (or without) the miR‐124 inhibitor(anti‐miR‐124). B‐F, Cell proliferation, cycle arrest, apoptosis, caspase‐3 and ‐ 9 activities were determined in Y79 cells after transfection with si‐NC and si‐NEAT1 with (or without) the anti‐miR‐124. *P < 0.05 and **P < 0.01. miR‐124, microRNA‐124; NEAT1, nuclear‐enriched abundant transcript 1; RB, retinoblastoma

To assess if NEAT1 exerts an oncogenic effect on RB cells via miR‐124, we performed a loss‐of‐function experiment and inhibited miR‐124 expression in Y79 cells with NEAT1 knockdown (Figure 4A). We found that miR‐124 inhibitor reversed the effect of NEAT1 depletion on cell proliferation, cycle arrest, apoptosis and caspase‐3 and ‐9 activities (Figure 5B‐F). These results imply that NEAT1 exerts its biological function in RB cells by partially regulating miR‐124.

## 4 DISCUSSION

Recently, several lncRNAs have been implicated in the onset and progression of cancers, including RB, and serve as diagnostic markers and therapeutic target for RB. For instance, Hu et al^21^ found that enhanced expression of XIST associates positively with an advanced stage of cTNM (III‐V), its late differentiation status, and that XIST promoted RB cell proliferation and decreased cell apoptosis via sponging miR‐124. Likewise, Yang et al^27^ reported aggravated RB through highly expressed HOTAIR by regulating miR‐613/c‐met axis and implied HOTAIR as a promising biomarker to be used in diagnostics and as a target for RB therapy. Bi et al^28^ showed the putative tumor‐suppressor role of MT1JP by negatively modulating the action of the Wnt/β‐catenin signaling pathway in the development of RB and that it may also be used as a prognostic biomarker and therapeutic target. Later, Li et al^29^ showed that LINC00152 knockdown significant impeded cellular activities associated with perpetuation, enhanced cell apoptosis and caspase‐3 and ‐9 activities in vitro, as well as suppressed tumorigenesis in vivo_._ In this study, we found increased expression of NEAT1 in RB‐affected tissues and cell lines. We also showed that NEAT1 knockdown impeded RB cell proliferation, cycle procession, and induced apoptosis through repression of miR‐124 expression by acting as a ceRNA. Hence, NEAT1 is a strong contender to act as a potential target for RB.

The expression of NEAT1 is upregulated and it functions as an oncogenic lncRNA in many human malignant cancers. However, its expression status, function, and mechanism of action in RB need to be unraveled. The current study is the first to our knowledge that has investigated NEAT1 function and mechanism of its action in RB. We first revealed that NEAT1 expression is upregulated in RB‐affected tissues and cell lines, and subsequently found that the knockdown of NEAT1 in RB cells remarkably inhibited cell proliferation, cycle progression, and promoted apoptosis, and caspase‐3 and ‐9 activities. These findings suggested an oncogenic role of NEAT1 in RB tumorigenesis.

LncRNAs functions as a ceRNA by harboring binding sites for miRNAs and decreasing miRNA expression.^25^ NEAT1 can act as ceRNAs for multiple miRNAs, such as miR‐196‐5p,^16^ miR‐211,^29^ miR‐384,^30^ miR‐342‐3p,^31^ miR‐506,^32^ and miR‐9‐5p.^33^ Therefore, we used the software StarBase2.0 (http://starbase.sysu.edu.cn) to search for miRNAs that could target NEAT1. Among miRNAs, miR‐124 was predicted as a target miRNA of NEAT1 based on its roles in RB. Studies report the downregulation of miR‐124 and its role as a tumor suppressor in several kinds of cancers including RB.^22^ Here, we confirmed through luciferase and RIP assays that NEAT1 indeed acts as a miR‐124 sponge in RB cells. We also found that NEAT1 downregulation remarkably enhances miR‐124 levels and that miR‐124 mimics significantly decrease NEAT1 expression, while inhibitor of miR‐124 obviously increased NEAT1 expression in Y79 cells. In addition, the miR‐124 expression is inversely related to the NEAT1 expression and was observed to be significantly downregulated in RB tissues and cells. Importantly, we also found that miR‐124 inhibitor reversed the effect of NEAT1 depletion on cell proliferation, cycle arrest, apoptosis and caspase‐3 and ‐9 activities. These results imply that NEAT1 exerts its role in RB cells by partially regulating miR‐124.

To conclude, the present study illustrates the oncogenic role of NEAT1 like that of an lncRNA to facilitate the tumorigenesis and RB progression by binding to miR‐124 competitively and suggest the potential of NEAT1 as a target for RB therapy. Since NEAT1 could regulate multiple miRNA or other target genes, thus, the findings of this study could pave the way to examine the underlying molecular mechanism of NEAT1 action.

## CONFLICT OF INTERESTS

The authors declare that there are no conflict of interests.

## AUTHOR CONTRIBUTION

Lufei Wang did all the experiments. Hui Zhang analyzed all data and was a major contributor in writing the manuscript. Defeng Yang and Rui Tian did some experimental work. All authors read and approved the final manuscript.

## References

[1] Chen W , Zheng R , Baade PD , et al. Cancer statistics in China, 2015. CA Cancer J Clin. 2016;66:115‐132. 26808342 10.3322/caac.21338

[2] Dimaras H , Kimani K , Dimba EA , et al. Retinoblastoma. Lancet. 2012;379:1436‐1446. 22414599 10.1016/S0140-6736(11)61137-9

[3] Zhang J , Benavente CA , McEvoy J , et al. A novel retinoblastoma therapy from genomic and epigenetic analyses. Nature. 2012;481:329‐334. 22237022 10.1038/nature10733 PMC3289956

[4] Peng WX , Koirala P , Mo YY . LncRNA‐mediated regulation of cell signaling in cancer. Oncogene. 2017;36:5661‐5667. 28604750 10.1038/onc.2017.184 PMC6450570

[5] Fatica A , Bozzoni I . Long non‐coding RNAs: new players in cell differentiation and development. Nat Rev Genet. 2014;15:7‐21. 24296535 10.1038/nrg3606

[6] Gutschner T , Diederichs S . The hallmarks of cancer: a long non‐coding RNA point of view. RNA Biol. 2012;9:703‐719. 22664915 10.4161/rna.20481 PMC3495743

[7] Fan Q , Yang L , Zhang X , et al. The emerging role of exosome‐derived non‐coding RNAs in cancer biology. Cancer Lett. 2018;414:107‐115. 29107112 10.1016/j.canlet.2017.10.040

[8] Shang W , Yang Y , Zhang J , Wu Q . Long noncoding RNA BDNF‐AS is a potential biomarker and regulates cancer development in human retinoblastoma. Biochem Biophys Res Commun. 2018;497:1142‐1148. 28131827 10.1016/j.bbrc.2017.01.134

[9] Zhang H , Zhong J , Bian Z , Fang X , Peng Y , Hu Y . Long non‐coding RNA CCAT1 promotes human retinoblastoma SO‐RB50 and Y79 cells through negative regulation of miR‐218‐5p. Biomed Pharmacother. 2017;87:683‐691. 28088735 10.1016/j.biopha.2017.01.004

[10] Yang Y , Peng XW . The silencing of long non‐coding RNA ANRIL suppresses invasion, and promotes apoptosis of retinoblastoma cells through ATM‐E2F1 signaling pathway. Biosci Rep. 2018;38:BSR20180558. 30355646 10.1042/BSR20180558 PMC6294646

[11] Bond CS , Fox AH . Paraspeckles: nuclear bodies built on long noncoding RNA. J Cell Biol. 2009;186:637‐644. 19720872 10.1083/jcb.200906113 PMC2742191

[12] Wang P , Wu T , Zhou H , et al. Long noncoding RNA NEAT1 promotes laryngeal squamous cell cancer through regulating miR‐107/CDK6 pathway. J Exp Clin Cancer Res. 2016;35:22. 26822763 10.1186/s13046-016-0297-z PMC4731996

[13] Bai YH , Lv Y , Wang WQ , Sun GL , Zhang HH . LncRNA NEAT1 promotes inflammatory response and induces corneal neovascularization. J Mol Endocrinol. 2018;61:231‐239. 30328354 10.1530/JME-18-0098

[14] Adriaens C , Standaert L , Barra J , et al. p53 induces formation of NEAT1 lncRNA‐containing paraspeckles that modulate replication stress response and chemosensitivity. Nat Med. 2016;22:861‐868. 27376578 10.1038/nm.4135

[15] Li X , Wang S , Li Z , et al. The lncRNA NEAT1 facilitates cell growth and invasion via the miR‐211/HMGA2 axis in breast cancer. Int J Biol Macromol. 2017;105:346‐353. 28720546 10.1016/j.ijbiomac.2017.07.053

[16] Zhong F , Zhang W , Cao Y , et al. LncRNA NEAT1 promotes colorectal cancer cell proliferation and migration via regulating glial cell‐derived neurotrophic factor by sponging miR‐196a‐5p. Acta Biochim Biophys Sin. 2018;50:1190‐1199. 30383193 10.1093/abbs/gmy130

[17] Qi L , Liu F , Zhang F , et al. lncRNA NEAT1 competes against let‐7a to contribute to non‐small cell lung cancer proliferation and metastasis. Biomed Pharmacother. 2018;103:1507‐1515. 29864936 10.1016/j.biopha.2018.04.053

[18] Xiong DD , Li ZY , Liang L , et al. The LncRNA NEAT1 accelerates lung adenocarcinoma deterioration and binds to Mir‐193a‐3p as a competitive endogenous RNA. Cell Physiol Biochem. 2018;48:905‐918. 30036873 10.1159/000491958

[19] Guo HM , Yang SH , Zhao SZ , Li L , Yan MT , Fan MC . LncRNA NEAT1 regulates cervical carcinoma proliferation and invasion by targeting AKT/PI3K. Eur Rev Med Pharmacol Sci. 2018;22:4090‐4097. 30024596 10.26355/eurrev_201807_15400

[20] Ling ZA , Xiong DD , Meng RM , et al. LncRNA NEAT1 promotes deterioration of hepatocellular carcinoma based on in vitro experiments, data mining, and RT‐qPCR analysis. Cell Physiol Biochem. 2018;48:540‐555. 30021196 10.1159/000491811

[21] Hu C , Liu S , Han M , Wang Y , Xu C . Knockdown of lncRNA XIST inhibits retinoblastoma progression by modulating the miR‐124/STAT3 axis. Biomed Pharmacother. 2018;107:547‐554. 30114638 10.1016/j.biopha.2018.08.020

[22] Liu F , Tai Y , Ma J . LncRNA NEAT1/let‐7a‐5p axis regulates the cisplatin resistance in nasopharyngeal carcinoma by targeting Rsf‐1 and modulating the Ras‐MAPK pathway. Cancer Biol Ther. 2018;19:534‐542. 29565706 10.1080/15384047.2018.1450119 PMC5927658

[23] Huang G , He X , Wei XL . lncRNA NEAT1 promotes cell proliferation and invasion by regulating miR365/RGS20 in oral squamous cell carcinoma. Oncol Rep. 2018;39:1948‐1956. 29484420 10.3892/or.2018.6283

[24] An J , Lv W , Zhang Y . LncRNA NEAT1 contributes to paclitaxel resistance of ovarian cancer cells by regulating ZEB1 expression via miR‐194. Onco Targets Ther. 2017;10:5377‐5390. 29180871 10.2147/OTT.S147586 PMC5691924

[25] Du Z , Sun T , Hacisuleyman E , et al. Integrative analyses reveal a long noncoding RNA‐mediated sponge regulatory network in prostate cancer. Nat Commun. 2016;7:10982. 26975529 10.1038/ncomms10982 PMC4796315

[26] Sun Y , Duan F , Liu W , et al. Comprehensive assessment of the relationship between MicroRNA‐124 and the prognostic significance of cancer. Front Oncol. 2018;8:252. 30062087 10.3389/fonc.2018.00252 PMC6055006

[27] Yang G , Fu Y , Lu X , Wang M , Dong H , Li Q . LncRNA HOTAIR/miR‐613/c‐met axis modulated epithelial‐mesenchymal transition of retinoblastoma cells. J Cell Mol Med. 2018;22:5083‐5096. 30030888 10.1111/jcmm.13796 PMC6156449

[28] Bi LL , Han F , Zhang XM , Li YY . LncRNA MT1JP acts as a tumor inhibitor via reciprocally regulating Wnt/beta‐catenin pathway in retinoblastoma. Eur Rev Med Pharmacol Sci. 2018;22:4204‐4214. 30024609 10.26355/eurrev_201807_15414

[29] Li S , Wen D , Che S , et al. Knockdown of long noncoding RNA 00152 (LINC00152) inhibits human retinoblastoma progression. Onco Targets Ther. 2018;11:3215‐3223. 29922070 10.2147/OTT.S160428 PMC5995430

[30] Zhu L , Yang N , Li C , Liu G , Pan W , Li X . Long noncoding RNA NEAT1 promotes cell proliferation, migration, and invasion in hepatocellular carcinoma through interacting with miR‐384. J Cell Biochem. 2018. 10.1002/jcb.27499 PMC6587825 30346062

[31] Wang L , Xia JW , Ke ZP , Zhang BH . Blockade of NEAT1 represses inflammation response and lipid uptake via modulating miR‐342‐3p in human macrophages THP‐1 cells. J Cell Physiol. 2019;234:5319‐5326. 30259979 10.1002/jcp.27340

[32] Yong W , Yu D , Jun Z , et al. Long noncoding RNA NEAT1, regulated by LIN28B, promotes cell proliferation and migration through sponging miR‐506 in high‐grade serous ovarian cancer. Cell Death Dis. 2018;9:861. 30154460 10.1038/s41419-018-0908-z PMC6113267

[33] Xie Q , Lin S , Zheng M , Cai Q , Tu Y . Long noncoding RNA NEAT1 promotes the growth of cervical cancer cells via sponging miR‐9‐5p. Biochem Cell Biol. 2019;97:100‐108. 30096244 10.1139/bcb-2018-0111

[34] Hu Y , Yang Q , Wang L , et al. Knockdown of the oncogene lncRNA NEAT1 restores the availability of miR‐34c and improves the sensitivity to cisplatin in osteosarcoma. Biosci Rep. 2018;38:BSR20180375. 29654165 10.1042/BSR20180375 PMC6435545

[35] Li Y , Chen D , Gao X , Li X , Shi G . LncRNA NEAT1 regulates cell viability and invasion in esophageal squamous cell carcinoma through the miR‐129/CTBP2 Axis. Dis Markers. 2017;2017. 5314649‐11 10.1155/2017/5314649 PMC5632864 29147064

[36] Liu S , Hu C , Wang Y , Shi G , Li Y , Wu H . miR‐124 inhibits proliferation and invasion of human retinoblastoma cells by targeting STAT3. Oncol Rep. 2016;36:2398‐2404. 27498908 10.3892/or.2016.4999

